# Diagnostic and treatment guidelines for thrombotic thrombocytopenic purpura (TTP) in Japan 2023

**DOI:** 10.1007/s12185-023-03657-0

**Published:** 2023-09-10

**Authors:** Masanori Matsumoto, Yoshitaka Miyakawa, Koichi Kokame, Yasunori Ueda, Hideo Wada, Satoshi Higasa, Hideo Yagi, Yoshiyuki Ogawa, Kazuya Sakai, Toshiyuki Miyata, Eriko Morishita, Yoshihiro Fujimura, For TTP group of Blood Coagulation Abnormalities Research Study Team, Research on Rare and Intractable diseases, Health and Labour Sciences Research Grants from the Ministry of Health, Labour and Welfare of Japan

**Affiliations:** 1 Department of Blood Transfusion Medicine and Department of Hematology, Nara Medical University, 840 Shijyo-cho, Kashihara, Nara 634-8521 Japan; 2 Department of Hematology, Saitama Medical University, Iruma, Japan; 3 Department of Molecular Pathogenesis, National Cerebral and Cardiovascular Center, Suita, Japan; 4 Department of Hematology/Oncology, Kurashiki Central Hospital, Kurashiki, Japan; 5 Department of General Medicine, Mie Prefectural General Medical Center, Yokkaichi, Japan; 6 Department of Hematology, Hyogo Medical University, Nishinomiya, Japan; 7 Department of Hematology and Oncology, Nara Prefecture General Medical Center, Nara, Japan; 8 Department of Hematology, Gunma University, Maebashi, Japan; 9 Department of Blood Transfusion Medicine, Nara Medical University, Kashihara, Japan; 10 Department of Cerebrovascular Medicine, National Cerebral and Cardiovascular Center, Suita, Japan; 11 Department of Clinical Laboratory Science, Kanazawa University, Kanazawa, Japan; 12 Department of Blood Transfusion Medicine, Nara Medical University, Kashihara, Japan

**Keywords:** TTP, ADAMTS13, TMA

## Abstract

Thrombotic thrombocytopenic purpura (TTP) can rapidly become a life-threatening condition, and the importance of its appropriate diagnosis and treatment cannot be overstated. Until recently, TTP has mainly been diagnosed by clinical findings such as thrombocytopenia and hemolytic anemia. In addition to these clinical findings, however, reduced activity of a disintegrin-like and metalloprotease with thrombospondin type 1 motif 13 (ADAMTS13) below 10% has become internationally accepted as a diagnostic criterion for TTP. TTP is classified as immune-mediated TTP (iTTP) if the patient is positive for anti-ADAMTS13 autoantibodies, and as congenital TTP (cTTP) if *ADAMTS13* gene abnormalities are detected. Fresh frozen plasma (FFP) transfusion is performed in patients with cTTP to supplement ADAMTS13. Plasma exchange therapy using FFP is conducted in patients with iTTP to supplement ADAMTS13 and to remove both anti-ADAMTS13 autoantibodies and unusually large von Willebrand factor (VWF) multimers. To suppress autoantibody production, corticosteroid therapy is administered in conjunction with plasma exchange. The monoclonal anti-CD-20 antibody rituximab is effective in patients with iTTP. In addition, caplacizumab, an anti-VWF A1 domain nanobody, has a novel mechanism of action, involving direct inhibition of platelet glycoprotein Ib–VWF binding. The recommended first-line treatments of iTTP in Japan are plasma exchange and corticosteroids, as well as caplacizumab.

## Principles underlying this document

Thrombotic thrombocytopenic purpura (TTP) is a serious condition that develops as a result of platelet aggregation and systemic microvascular thrombosis [1]. Along with hemolytic uremic syndrome (HUS), TTP is a typical example of thrombotic microangiopathy (TMA). TTP is characterized as either congenital TTP [cTTP or Upshaw-Schulman syndrome (USS)] or immune-mediated TTP (iTTP, previously acquired TTP). Previously, due to the absence of specific diagnostic markers, TTP was diagnosed primarily based on the classic pentad of thrombocytopenia, hemolytic anemia, renal dysfunction, fever, and neurologic deficits. However, recent studies have demonstrated the association between its pathogenesis and the reduced activity of a disintegrin-like and metalloprotease with thrombospondin type 1 motifs 13 (ADAMTS13) [2, 3]. This enzyme specifically cleaves von Willebrand factor (VWF), a glycoprotein necessary for normal hemostasis. Consequently, decreased ADAMTS13 activity has been included in the diagnostic criteria for TTP [4]. cTTP is attributable to *ADAMTS13* gene mutations [5, 6], whereas iTTP develops as a result of anti-ADAMTS13 autoantibody production [2, 3].

In 2017, the expert physicians who were members of the TTP group of the Blood Coagulation Abnormalities team in the Japanese government-funded Health, Labour, and Welfare Scientific Research Grant Project published their consensus report entitled "Diagnostic and Treatment Guidelines for TTP 2017” [7]. Given the improvements in the national health insurance policy for TTP, i.e., coverage of ADAMTS13 activity and inhibitor assays and removal of the upper frequency limit of plasma exchange treatments, the original guidelines were partially revised and published as the “Diagnostic and Treatment Guidelines for TTP 2020” (Japanese version only). The present article is an update to the 2020 guidelines, and includes key clinical question (CQs) and answers regarding rituximab according to the "Medical Information Network Distribution Service (MINDS) Manual for Clinical Practice Guideline Development (https://minds.jcqhc.or.jp/english/s/project_overview).

Given the rarity of this disease, the authors acknowledge the difficulty of reviewing a large population of TTP patients. Nonetheless, we seek to provide the best possible evidence-based description of this condition. In the present guidelines, separate sections are devoted to iTTP and cTTP, and treatment recommendations are sorted by different levels of evidence according to the Grading of Recommendations, Assessment, Development, and Evaluation (GRADE) system (Table 1). Treatments for iTTP relate only to adult patients, and attention should be paid to pharmacotherapies for pediatric TTP patients due to the lack of clinical experience in this population. Patients whose ADAMTS13 activity is ≥ 10% are outside the scope of these guidelines, because such patients do not meet the criteria for designated intractable/rare diseases defined by the Japanese Ministry of Health, Labour, and Welfare (MHLW) (ADAMTS13 activity < 10% for TTP). In a certain group of patients, the presence of the classic pentad of signs strongly suggests TTP despite no marked reductions in ADAMTS13 activity. Further research must be performed to gain a better understanding of the pathology in such cases. As in people who meet the TTP criteria, plasma exchange and other appropriate treatments should be considered in these patients without delay.

**Table 1:** Levels of recommendations based on the GRADE system

Strength of recommendation
1. Strong
Benefits clearly outweigh risks and burden (or vice versa) in most patients
2. Weak
Benefits are closely balanced with risks and burden
Quality of supporting evidence
A: Evidence established from multiple RCTs or very strong evidence from observational studies
B: Limited evidence from RCTs or strong evidence from observational studies
C: Evidence from RCTs with serious flaws or weak or indirect evidence from observational studies

*GRADE* grading of recommendations assessment, development, and evaluation;* RCT* randomized controlled trial

## Pathology

ADAMTS13 is the 13th member of the ADAMTS family of metalloproteases [8]. VWF, the substrate of ADAMTS13, is mainly produced in vascular endothelial cells. In the plasma, unusually large VWF multimers (UL-VWFMs) are released and readily undergo ADAMTS13 proteolysis [1]. The platelet-binding affinity of VWF multimers is dependent on their molecular weight. Thus, UL-VWFMs are more likely to form platelet thrombi than normal VWF multimers [9]. Platelet–VWF interaction and adhesion are facilitated by high shear stress [10], which develops in the presence of fast blood flow and in small vessels. In patients with TTP, endothelial UL-VWFMs secreted into the bloodstream tend to remain uncleaved due to low ADAMTS13 activity. In microvessels, exposure to high fluid shear stress induces a conformational change in VWF, which leads to platelet adhesion and subsequent thrombosis. Platelet thrombosis in the microvasculature causes damage to the kidneys, brain, and other end organs.

## ADAMTS13 testing

Initially, ADAMTS13 proteolytic activity was measured using a full-length VWF as a substrate [11, 12]. Because this method is time consuming, recent assays employ shorter synthetic derivatives [13], yielding results in several hours [14, 15]. Results are typically reported in units or percentage relative to the measurements in pooled normal plasma, with 1 U/mL or 100% representing the normal levels of ADAMTS13 activity.

There are two known types of anti-ADAMTS13 autoantibodies: neutralizing (inhibitor) and non-neutralizing (binding) autoantibodies [16]. The former inhibits the protease in vitro, whereas the latter binds to but does not suppress it. Anti-ADAMTS13 autoantibodies are mostly inhibitory immunoglobulin G (IgG), with a minor proportion of IgA or IgM [17]. In Japan, it is common to perform Bethesda assays for only inhibitory ADAMTS13 autoantibodies, and non-neutralizing antibodies are primarily measured for research purposes. The amount of inhibitor is typically reported in Bethesda units (BU), with 1 BU representing 50% inhibition of ADAMTS13 activity in a one-to-one mixture of the test and normal plasma samples [18]. The majority of patients with iTTP have demonstrable levels of inhibitors [19, 20]. However, low inhibitor titers, i.e., < 1 BU/mL, do not constitute convincing evidence for the diagnosis of iTTP. As described below, patients who are negative for ADAMTS13 inhibitors may nevertheless have iTTP involving autoantibody-mediated ADAMTS13 deficiency. Such patients have non-neutralizing autoantibodies, the presence of which is suggested by their clinical manifestations.

In Japan, ADAMTS13 activity and inhibitor tests have been covered by the national health insurance since 2018. At many hospitals that outsource these tests to contract laboratories, it often takes 3 to 5 days before the results become available. As a tool to quickly predict acquired ADAMTS13 deficiency, the French scoring system [21] and the PLASMIC scoring system [22] have been developed for clinical use (Table 2) [23]. Likelihoods of severe deficiency of ADAMTS13 activity (< 10%) in high risk group in the French and PLASMIC scoring systems are 94% (2 points) and 62–82% (6–7 points), respectively. These two models predict ADAMTS13 deficiency in patients with suspected TMA, and are not designed for asymptomatic patients.

**Table 2:** French and PLASMIC scores

Parameters	French score	PLASMIC score
Platelet count	< 30 × 10^9^/L (+ 1)	< 30 × 10^9^/L (+ 1)
Serum creatinine	< 2.26 mg/dL (+ 1)	< 2.0 mg/dL (+ 1)
Hemolysis		+ 1
Indirect bilirubin > 2 mg/dL or reticulocyte count > 2.5% or undetectable haptoglobin		
No active malignancy in previous year		+ 1
No history of solid organ or stem cell transplantation		+ 1
PT-INR < 1.5		+ 1
MCV < 90 fL		+ 1
Likelihood of severe decrease in ADAMTS13 activity (< 10%)	0: 2%	0–4: 0–4%
	1: 70%	5: 5–24%
	2: 94%	6–7: 62–82%

The French and PLASMIC scores range from 0–2 to 0–7, respectively

These two scoring systems are used in patients suspected of having thrombotic microangiopathy. This table was adapted from reference [23]

*PT-INR* prothrombin time-international normalized ratio;* MCV* mean corpuscular volume;* ADAMTS13* a disintegrin-like and metalloproteinase with thrombospondin type 1 motifs 13

## Immune-mediated TTP

### Historical background

In 1924, the American physician Eli Moschcowitz documented the first presumed case of iTTP [24], and a series of similar cases without clear etiology were reported thereafter. In 1966, Amorosi and Ultmann described five common signs and symptoms [25], which are now referred to as the classic TTP pentad. Among them, the diagnostic importance of thrombocytopenia and hemolytic anemia gained increasing attention, and these conditions underscore the utility of plasma exchange [26]. Singer and colleagues were the first to use the term TTP in their 1947 publication [27].

Whereas the clinical benefit of plasma exchange for treating TTP was demonstrated in 1991, the pathogenetic mechanism of TTP remained unknown. In the early 1980s, findings of UL-VWFMs in the plasma of TTP patients [28] and VWF-rich platelet thrombi at autopsy [29] suggested the involvement of VWF. Following the establishment of the assay method for VWF protease (ADAMTS13) activity in 1996 [11, 12], patients with TTP were reported in 1998 to show severely reduced ADAMTS13 activity [2, 3]. However, given that the diagnosis of iTTP is not necessarily associated with a marked reduction in ADAMTS13 activity [30–32], ADAMTS13 deficiency and clinical manifestations remained as distinct diagnostic criteria. Physicians have debated the diagnostic threshold for ADAMTS13 activity reduction. There is growing international consensus on the use of activity levels < 10% to support the diagnosis of TTP [33].

### Definition

iTTP is caused by the systemic microvascular aggregation of platelets as a result of autoantibody-mediated severe ADAMTS13 deficiency.

### Epidemiology

Overseas, the annual incidence rate of clinically diagnosed TTP has been estimated at 4–11 per million population [34, 35]. The incidence based on ADAMTS13 measurement (< 10% activity) is unknown. One group of researchers reported an incidence of 1.74 per million population using a diagnostic criterion of ADAMTS13 activity < 5% [34]. In overseas studies of TTP based on ADAMTS13 activity, the median age at onset ranged from 36 to 51 years, with the proportion of females ranging from 65 to 100% [19, 31, 36, 37]. In a large study of Japanese patients with ADAMTS13-deficient TTP (ADAMTS13 activity < 5%), the median age at onset was 54 years, with a female proportion of 55% [20].

### Diagnostic algorithm

If a patient presents with reduced platelet count and hemolytic anemia of unknown cause, the clinician should measure ADAMTS13 activity. If the activity level is < 10%, the patient is diagnosed with TTP. If the patient is positive for anti-ADAMTS13 autoantibodies, the patient is diagnosed with iTTP. Clinicians should be aware that the patient may present with anti-ADAMTS13 autoantibodies even if they are negative for inhibitory autoantibodies. If the patient has no underlying disorders, they are diagnosed with primary iTTP. If anti-ADAMTS13 autoantibodies are produced secondary to systemic lupus erythematosus or other autoimmune diseases, or in response to ticlopidine or similar drugs, the diagnosis is secondary iTTP. When it takes several days to obtain the result of ADAMTS13 testing, the French scoring system [21] and the PLASMIC scoring system can be applied to predict the possibility of ADAMTS13 deficiency. In case of high risk of TTP, preemptive plasma exchange and corticosteroid therapy must be initiated immediately.

#### General laboratory thresholds related to the classic TTP pentad

##### Thrombocytopenia

For the diagnosis of thrombocytopenia, the general upper limit of the platelet count is 100 × 10^9^/L. Most patients with TTP have platelet counts in the range of 10 to 30 × 10^9^/L. The median platelet count in a cohort of Japanese patients with TTP was 10 × 10^9^/L [20].

##### Hemolytic anemia

Patients with iTTP exhibit microangiopathic hemolytic anemia, characterized by mechanical damage to red blood cells. Hemoglobin levels are typically in the range of 8–10 g/dL in overseas patients with TTP [38], whereas the median value in Japanese TTP patients was reported to be 7.3 g/dL [20]. The diagnosis of hemolytic anemia is made based on the following: the presence of schistocytes; increased levels of indirect bilirubin, lactate dehydrogenase, and reticulocytes; significantly decreased levels of haptoglobin; and a negative direct Coombs test. Peripheral smears showing a schistocyte percentage > 1% in the absence of additional severe erythrocyte shape abnormalities are robust indicators of the presence of TMA [39]. However, clinicians should be careful in regarding schistocytes as the hallmark of the disease, as quantifying the morphologic changes of blood cells is technically challenging and schistocytes may not always be present in the blood samples collected from patients with TTP.

##### Renal impairment

Renal failure may present as mild changes involving only occult blood or positive urinary protein, while more severe cases may be associated with elevated serum creatinine levels. In most cases, the serum creatinine levels are < 2 mg/dL [20]. Severe acute renal dysfunction that requires immediate hemodialysis is suggestive of HUS.

##### Fever

Patients with TTP may present with a mild (37–39 °C) or high (> 39 °C) fever. Fever is documented in approximately 30% [36, 37] to 72% [20] of patients with TTP.

##### Fluctuating neurologic conditions

Patients with TTP may develop a variety of neurologic conditions, ranging from mild headache to more severe manifestations such as delirium, confusion, personality change, decreased level of consciousness, quadriplegia, convulsions, etc. Neurologic manifestations usually fluctuate in severity and location, and often improve dramatically following plasma exchange. Neurologic involvement was reported in 79% of Japanese patients [20], and several overseas studies reported a rate of approximately 50% [19, 31].

## Differential diagnosis

The diagnostic criterion based on ADAMTS13 activity provides a clear guidance for differentiating common disorders from TTP, as described below. TMAs are subclassified according to their etiology. These etiology-based subclassifications correspond to clinical diagnoses, as shown in Table 3.

**Table 3:** Etiology-based subclassifications and clinical diagnoses of TMA

Etiology-based subclassification	Etiology	Underlying cause	Clinical diagnosis	Important clinical findings
ADAMTS13-deficient TMA	Severe decrease in ADAMTS13 activity	*ADAMTS13* gene abnormality	Congenital TTP (Upshaw-Schulman syndrome)	*ADAMTS13* gene abnormality
		Anti-ADAMTS13 autoantibodies	Immune-mediated TTP	Severe decrease in ADAMTS13 activity and the presence of anti-ADAMTS13 autoantibodies
Infection-induced TMA	Infection	STEC (e.g., *Escherichia coli* O157)	STEC-HUS	STEC infection established by blood or stool culture
		Neuraminidase-secreting *Streptococcus pneumoniae*	Pneumococcal-associated HUS	Proven pneumococcal infection
Complement-mediated TMA	Complement abnormality	Hereditary complement abnormalities (e.g., factors B, H, and I; C3; and membrane cofactor protein)	Atypical HUS	Genetic complement factor abnormalities; Low C3 and normal C4 levels (not necessarily observed in all patients with atypical HUS)
				Proven presence of anti-factor H antibodies
		Anti-factor H antibodies		
Coagulation-mediated TMA	Coagulation abnormality	Mutations in diacylglycerol kinase ε and thrombomodulin genes	Atypical HUS (possibly)	Proven genetic mutations
Secondary TMA	Unknown	Autoimmune diseases	Connective tissue disease–associated TMA, etc	SLE, scleroderma, or other connective tissue disorders
		Hematopoietic stem cell transplant	Hematopoietic stem cell transplantation–associated TMA	Unresponsive to platelet transfusion Hemolysis (accompanied with, e.g., low haptoglobin levels)
		Organ transplant (e.g., kidney, liver)	Post–organ transplant TMA	Thrombocytopenia of unknown etiology and hemolysis (accompanied with, e.g., low haptoglobin levels)
		Malignant tumors	Tumor-associated TMA	Frequently diagnosed in patients with malignant lymphomas, stomach cancer, and pancreatic cancer
		Pregnancy	Pregnancy-associated TMA, HELLP syndrome	HELLP syndrome typically develops at ≥ 30 weeks of gestation in combination with hypertension
		Drugs (e.g., mitomycin)	Drug-induced TMA	Medication prescription
Other TMAs	Unknown	Other	TTP-like disorders or similar	Classic TTP pentad

*TMA* thrombotic microangiopathy; *TTP* thrombotic thrombocytopenic purpura; *HUS* hemolytic uremic syndrome; *SLE* systemic lupus erythematosus; *THBD* thrombomodulin; HELLP syndrome, hemolysis, elevated liver enzymes, and low platelet count syndrome

### Disseminated intravascular coagulation

In patients with TTP, prothrombin time (PT) and activated partial thromboplastin time (APTT) are normal, fibrinogen and antithrombin levels typically remain in the normal range, and fibrin/fibrinogen degradation products (FDP) and D-dimer levels are mildly elevated [40]. In contrast, disseminated intravascular coagulation (DIC) is usually characterized by fibrin thrombi, prolonged PT and APTT times, and reduced levels of fibrinogen.

### Hemolytic uremic syndrome

HUS induced by Shiga toxin–producing *Escherichia coli* (STEC) strains, such as *E. coli* O157, can be diagnosed by using (a) stool culture, enzyme immunoassay (EIA), and other methods for detecting STEC, or (b) methods for detecting anti-lipopolysaccharide (LPS) IgM antibodies. Formerly, cases of HUS unrelated to STEC infection were referred to as atypical HUS (aHUS). More recently, the term aHUS has been used to refer only to HUS involving complement abnormalities, such as complement factor H, component 3 (C3), and other elements [41], and is classified etiologically as a complement-mediated TMA.

### HELLP syndrome

HELLP (hemolysis, elevated liver enzymes, and low platelet count) syndrome occurs in connection with pregnancy-induced hypertensive nephropathy or preeclampsia, and can lead to multiple organ failure. The diagnostic criteria proposed by Sibai et al. are commonly used [42], but these criteria are not helpful for discriminating between HELLP syndrome and TTP. Patients with severely reduced ADAMTS13 activity < 10% of normal should be diagnosed with TTP.

### Evans syndrome

Evans syndrome is defined as the combination of autoimmune hemolytic anemia and immune thrombocytopenia (ITP). Patients with Evans syndrome demonstrate a positive direct Coombs test. Certain patients diagnosed with Evans syndrome are direct Coombs–negative, and this group may include individuals who should be diagnosed with TTP based on severely decreased ADAMTS13 activity.

### Secondary thrombotic microangiopathy

TMAs are reported to develop secondary to the following: systemic lupus erythematosus, scleroderma, and other connective tissue diseases; transplantation of hematopoietic stem cells, kidneys, and other organs; and malignant diseases. Although the exact etiology of secondary TMA is unknown, many scientists believe that it is primarily attributable to endothelial cell injury.

### Other thrombotic microangiopathies

Some patients who are clinically diagnosed with TMA in the absence of underlying conditions do not satisfy the TTP criterion of a severe decrease in ADAMTS13 activity to < 10%. This group of patients includes those who were formerly diagnosed with TTP based on the classic pentad.

## Treatments

Plasma exchange is the only historically accepted treatment modality for iTTP [26]. In light of a report indicating that a longer delay in initiating plasma exchange is an independent predictor of treatment failure [43], the clinician should start plasma exchange as soon as possible if the patient is suspected of having iTTP. Plasma exchange should be implemented before the ADAMTS13 assay report is finalized, since final results may not be available for several days after the assay is ordered. Caplacizumab, a single-chain humanized monoclonal antibody against the A1 domain of human VWF, was approved in September 2022 for marketing in Japan [44]. This drug has a novel mechanism of action, specifically the direct inhibition of platelet glycoprotein (GP) Ib–VWF binding, and controlled double-blind trials demonstrated that it reduced the mortality of iTTP patients; notably, this was the first time that this mortality was reduced by a modality other than plasma exchange [45].

The following sections describe current treatment recommendations and the grades for the strength of the underlying evidence (Table 1).

### Acute phase

#### Plasma exchange (grade of recommendation: 1A)

The physician should carry out plasma exchange with fresh frozen plasma (FFP) once daily. The FFP volume is 1.0–1.5 times the patient’s circulating plasma volume, which can be estimated using the equation below. The effectiveness of plasma exchange is attributable to: (1) ADAMTS13 supplementation, (2) removal of ADAMTS13 inhibitors, and (3) elimination of UL-VWFMs unsusceptible to proteolytic cleavage [32]. Human serum albumin solutions are not recommended for replacement, because these do not supplement ADAMTS13. FFP infusion may be performed in emergency cases. An analysis of response and survival showed that plasma exchange is superior to plasma infusion in the treatment of TTP [26].

$${\text{Circulating plasma volume }}\left( {{\text{mL}}} \right)\, = \,{\text{body weight }}\left( {{\text{kg}}} \right)\, \times \,{7}0 \, \left( {{\text{mL}}/{\text{kg}}} \right)\, \times \,\left( {{1}\, - \,{\text{hematocrit value }}\left[ \% \right]/{1}00} \right).$$

Plasma exchange

The physician should perform plasma exchange with FFP (50–75 mL/kg) once daily. It should be continued until 2 days after the platelet count is normalized (≥ 150 × 10^9^/L). Previously, the Japanese national health insurance policy restricted the frequency of plasma exchange treatments to three times per week. In April 2018, the same plasma exchange regimen as that recommended in the British Committee for Standards in Haematology guidelines was approved [38].

#### Corticosteroid therapy (grade of recommendation: 1B)

Corticosteroids are administered either as pulse or high-dose therapy. Available data are inconclusive regarding the superiority of either modality [46]. Corticosteroids are expected to suppress autoantibody production. The physician should consider lower doses in patients who are elderly or have diabetes mellitus or severe infections. Although corticosteroid use in patients with TTP is considered off-label under current Japanese health insurance standards, it is classified as a Grade-1 recommendation based on its popularity in routine clinical practice.

Pulse corticosteroid therapy (off-label use in Japan)

Methylprednisolone 1000 mg

After receiving plasma exchange, the patient is administered methylprednisolone 1000 mg once daily over approximately 2 h by drip infusion. The dose should be tapered after administration for 3 consecutive days. The dose-reduction regimen is made with reference to the platelet count and ADAMTS13 test results. No evidence-based procedure has been established for corticosteroid dose reduction. If the physician wishes to extend methylprednisolone therapy for the treatment of neurologic symptoms, or under conditions of intensive care unit (ICU) management, the corticosteroid may be given at 500, 250, and 125 mg/day, with each dosage administered for 2 days and in this order. The dosage regimen should then be switched to oral corticosteroids 30 mg/day, and subsequently tapered with reference to the description below. If the physician decides to switch to oral therapy after the first 3 days of drip infusion, oral prednisolone 0.5–1.0 mg/kg/day should be administered and the dosage tapered based on the description below.

High-dose corticosteroid therapy (off-label use in Japan)

Oral prednisolone 1 mg/kg/day

The physician tapers the prednisolone dosage with reference to the platelet count and ADAMTS13 test results. The initial dosage (1 mg/kg/day) is maintained for 2 weeks, followed by a rapid reduction to 0.5 mg/kg/day. The physician should further reduce the total weekly dose by approximately 2.5–5.0 mg each week, taking note of the platelet count and ADAMTS13 inhibition test results.

#### Caplacizumab (grade of recommendation: 1A)

Caplacizumab is a small molecule antibody that directly inhibits the thrombus formation process mediated by platelet–VWF interaction [47, 48]. In overseas randomized double-blind trials, caplacizumab significantly reduced the time to platelet count normalization when administered concomitantly with plasma exchange and corticosteroid therapy [47, 48]. An open-label phase II/III trial has been completed in Japan [44].

Caplacizumab therapy

Caplacizumab 10 mg

On the first day, a 10-mg dose is administered intravenously at least 15 min before the start of plasma exchange, and another 10-mg dose is administered subcutaneously after the end of the plasma exchange session. On subsequent days, a 10-mg dose is administered subcutaneously after each daily plasma exchange. After termination of the plasma exchange therapy, once-daily 10-mg doses are administered subcutaneously for 30 days. If ADAMTS13 activity remains < 10% after the 30-day regimen, caplacizumab may be continued for an additional 28 days. Although caplacizumab therapy may be started before a marked decrease in ADAMTS13 activity is confirmed, it should be discontinued immediately if ADAMTS13 activity is found to be ≥ 10% and TTP is ruled out.

#### Rituximab (grade of recommendation: 2B)

Rituximab is an anti-human CD20 chimeric monoclonal antibody that suppresses ADAMTS13 inhibitor production by depleting B lymphocytes. This agent is typically administered for treating patients with refractory TTP [49–51]. In Japan, rituximab is indicated for refractory or recurrent TTP only, and its use in the acute phase of TTP is not subject to national health insurance coverage. However, physicians may consider rituximab for the treatment of patients in the acute phase of iTTP (see CQ1 below).

Rituximab therapy

Rituximab 375 mg/m^2^

Rituximab may cause serious adverse reactions, including infusion reactions (e.g., pyrexia, decreased blood pressure, urticaria, and hypoxemia). Therefore, premedications (antihistaminic drugs and acetaminophen) should be administered, and an infusion pump should be employed to increase the infusion rate gradually. The physician should use particular caution in cases of patients receiving rituximab for the first time. In patients undergoing plasma exchange, rituximab should be administered after the end of the plasma exchange session.

The recommended rituximab therapy regimen is once weekly for 4 weeks.

#### Antiplatelet drugs (grade of recommendation: 2B)

Antiplatelet drugs can be effective in the treatment of TTP because platelet thrombi formed via platelet GP 1b–VWF interaction cause TTP. In an Italian randomized study of acute-phase TTP, add-on treatment with aspirin and dipyridamole did not significantly improve the response rate, but did decrease mortality [52]. It should be noted that in one small-scale study of acute-phase TTP patients, the use of aspirin and dipyridamole increased the risk of serious bleeding complications [53]. Moreover, given that iTTP developed in association with ticlopidine and clopidogrel [54, 55], these drugs should be avoided in patients with TTP. Low-dose aspirin is empirically administered in patients whose platelet count has returned to > 50 × 10^9^/L [38], although its impact on preventing TTP relapse has not been substantiated. However, concomitant use of aspirin and caplacizumab may increase the risk of bleeding and should be avoided.

Antiplatelet pharmacotherapy (off-label use in Japan)

Oral aspirin may be administered at 81–100 mg once daily in the morning until corticosteroids are terminated.

#### Other treatments

Patients without cardiac disorders may undergo red blood cell transfusion if hemoglobin levels decrease to < 7.0 g/dL [38, 56] (grade of recommendation: 1A). In patients with cardiac disorders, hemoglobin levels < 8.0 g/dL may suggest the need for red blood cell transfusion [57]. Platelet transfusion is indicated for patients with life-threatening bleeding. Prophylactic platelet transfusion in other situations, however, is contraindicated as it may aggravate thrombosis [38, 58, 59] (grade of recommendation: 1B).

In patients manifesting iTTP secondary to the use of a specific drug, the drug should be immediately discontinued. If the patient has an underlying condition, only the drugs to treat the condition should be continued. Since ADAMTS-13 activity levels may be severely lowered in patients with secondary iTTP, plasma exchange in these patients should be conducted using a similar protocol as that used in patients with primary TTP.

### Refractory or early-relapse TTP

If the platelet count does not increase to 50 × 10^9^/L after five plasma exchange sessions, or if the platelet count decreases to < 50 × 10^9^/L after initially recovering to > 150 × 10^9^/L, the physician should consider rituximab in combination with plasma exchange [51] (grade of recommendation: 1B). Preferably, the physician should order ADAMTS13 activity and inhibitor assays at multiple time points because repeated plasma exchange sessions may boost ADAMTS13 inhibitor titers [60]. Japan's national health insurance policy allows for once-weekly testing of ADAMTS13 activity and inhibitor levels within 1 month of the diagnosis of TTP. It allows for an additional 4 weeks of testing for patients undergoing caplacizumab therapy. Patients with elevated ADAMTS13 inhibitor levels are often unresponsive to plasma exchange alone, thereby necessitating concurrent use of rituximab. Rituximab takes 10–14 days to demonstrate a clinical impact, and plasma exchange should often be conducted in the interim.

#### Rituximab (grade of recommendation: 1B)

Rituximab is recommended for the treatment of recurrent or refractory TTP because multiple open-label Phase II trials have confirmed the efficacy and safety of rituximab for this indication (see CQ2 below). No randomized controlled studies have been conducted to evaluate rituximab for this indication. This therapy is covered by the Japanese national health insurance system.

Therapies other than rituximab can often (but not always) be effective for treating patients with refractory or recurrent TTP. The following therapies are representative and not exhaustive:

#### Cyclophosphamide (off-label use in Japan, grade of recommendation: 2B) [61]

Cyclophosphamide 500 mg/m^2^

Cyclophosphamide is administered once daily over 2 h. Typically, the cyclophosphamide regimen is restricted to a single dose, as multiple doses may cause bone marrow suppression.

#### Vincristine (off-label use in Japan, grade of recommendation: 2B) [62]

Vincristine 1 mg/m^2^ is administered intravenously once daily at a slow infusion rate. The vincristine regimen is usually restricted to a single dose, as multiple doses may cause neurotoxicity and bone marrow suppression.

#### Cyclosporine (off-label use in Japan, grade of recommendation: 2B) [63]

Oral cyclosporine 4 mg/kg/day is administered in two divided doses.

Blood levels of cyclosporine should be monitored to maintain a trough level of approximately 100–200 ng/mL.

#### Other treatments (off-label use in Japan)

Formerly, splenectomy (grade of recommendation: 2C) [64] and high-dose immunoglobulin (grade of recommendation: 2C) [65] were frequently administered to treat patients with refractory or recurrent TTP. These treatments have been superseded by rituximab therapy.

### Remission period

After the patient has achieved full remission, the physician should discontinue corticosteroids at the earliest opportunity based on ADAMTS13 activity and inhibitor titer measurements. No effective prophylactic treatments have been recommended for patients in remission. To minimize the risk of relapse, patients should visit the physician regularly during the first several years after achieving remission and undergo monitoring of the platelet count and ADAMTS13 activity. In remitted patients with normal platelet counts, ADAMTS13 activity may be severely reduced and ADAMTS13 inhibitors may be present [17, 66]. Patients whose ADAMTS13 activity is significantly reduced (ADAMTS13 relapse) or who are positive for ADAMTS13 inhibitors are at high risk of clinical relapse [17, 66]. The preemptive use of rituximab is a viable option. If patients in remission of iTTP show an ADAMTS13 activity decrease to < 10%, rituximab may be considered for preventing relapse (off-label use in Japan, see CQ3 below).

### Classification by severity

Table 4 summarizes the TTP severity classification scheme established by the Japanese MHLW for a medical expense support system and used in designated intractable/rare diseases. If the patient’s condition meets one or more of these eight criteria, the condition is classified as moderate or severe, and the patient is eligible for the designated intractable/rare disease program.

**Table 4:** iTTP severity classification scheme

1. ADAMTS13 inhibitor titer ≥ 2 BU/mL
2. Renal failure
3. Neurologic dysfunction
4. Cardiac manifestations (elevated troponin levels, abnormal ECG findings, etc.)
5. Gastrointestinal symptoms (e.g., abdominal pain)
6. Deep-vessel hemorrhage or thrombosis
7. Treatment resistance
8. Recurrence
Criteria and Evaluation: Add 1 point for each “yes” response (0 points for “no”)
Severe: ≥ 3
Moderate: 1–2
Mild: 0

### Treatment outcome

TTP was formerly associated with a poor prognosis, with a mortality rate > 90% in untreated patients [25]. The introduction of plasma exchange, however, raised the survival rate to approximately 80% [19, 20, 26, 31, 36]. High levels of serum creatinine and ADAMTS13 inhibitor titers of ≥ 2 BU/mL are poor prognostic factors in patients with ADAMTS13 activities < 10% [19]. Patients with acute TTP are at risk of cardiovascular death, and should undergo myocardial troponin measurement [38]. Attention should be paid to the risk of sudden death if high troponin levels suggest ischemic heart disease.

## Congenital TTP (USS)

### Historical background

Neonatal cases of TTP characterized by severe jaundice and thrombocytopenia were documented in the early 1950s [67]. In 1960, Schulman and colleagues reported an 8-year-old girl who experienced repeated bleeding events associated with chronic thrombocytopenia beginning in the neonatal period [68]. One key clinical characteristic of this patient was that her platelet counts markedly improved following transfusion of small amounts of FFP. In 1978, Upshaw reported a similar case of a 29-year-old woman with chronic thrombocytopenia who also responded well to FFP transfusion [69]. These conditions were referred to as USS [70], and low blood fibronectin levels were associated with its etiology, although this association was later refuted. Thereafter, the term USS was abandoned in the Western medical community and superseded by “chronic-relapsing (CR) TTP” [28]. While the latter term gained lasting popularity, it obscured the fact that TTP can be differentiated into congenital and acquired types.

In 1982, Moake et al. made the important discovery that UL-VWFMs appear in remitted patients with CR-TTP [28]. In 1997, Furlan et al. reported that VWF-cleaving protease (CP) activity is severely reduced in patients with CR-TTP [71]. The VWF-CP was later identified as ADAMTS13. The CR-TTP patient cohorts in these studies included individuals with congenital and iTTP. Furlan et al. reported that the parents of the two sibling patients in the study had essentially normal VWF-CP activity. In a study of three Japanese USS patients reported in July 2001 [72], Kinoshita et al. showed that the VWF-CP activities of the patients were undetectable, while those of their parents ranged from 5.6% to 60% relative to control values. The authors concluded that USS is inherited in an autosomal recessive fashion. Levy et al. conducted genome-wide positional cloning in four pedigrees of individuals with cTTP, and reported in December 2001 that *ADAMTS13* was the responsible gene [5]. These findings led to the recognition that USS is a congenital form of TTP arising from *ADAMTS13* gene abnormalities, and thus the terms “USS” and “congenital TTP” are synonyms. Two rapid assays for determining ADAMTS13 activity were recently developed in Japan [14, 15], and these assays accelerated the diagnosis of USS patients.

### Definition

cTTP is a hereditary disorder resulting from systemic microvascular platelet aggregation, and is attributable to *ADAMTS13* gene mutations causing severe ADAMTS13 deficiency. Its inheritance is autosomal recessive.

### Epidemiology

The prevalence rate of cTTP is unknown. The population of patients with cTTP is assumed to be much smaller than that of patients with iTTP [73]. One European registry-based study estimated that the prevalence was in the range of 0.5–4 per million population [74]. A group of Japanese researchers estimated that this disease develops in 1 per 1.1 million population [75]. By the end of 2022, 70 patients with cTTP were identified in Japan. Since this disease exhibits autosomal recessive inheritance, it should theoretically develop at equal rates in both sexes. However, the group of 70 patients documented in Japan consists of 29 males and 41 females. The larger number of female patients with cTTP may be attributable to the greater chance of detection during pregnancy.

Depending on the timing of onset, this disease can be subcategorized into two types [76]:

*Early onset*: typically, 25–40% of patients with early-onset cTTP develop severe Coombs-negative jaundice in their neonatal period that requires exchange blood transfusion [77]. Although their neonatal jaundice is generally accompanied by thrombocytopenia, their platelet count may not always fall below 50×10^9^/L [77]. During infancy or childhood, thrombocytopenic episodes frequently occur during febrile infections. These manifestations may often lead to the misdiagnosis of ITP. Periodic transfusion of FFP is indicated for patients diagnosed with cTTP.

*Late onset*: patients may be diagnosed with cTTP in their adolescence or adulthood after experiencing thrombocytopenia in association with pregnancy, influenza, or other infections. Some patients who experience late-onset TTP may have been misdiagnosed with ITP during childhood because of thrombocytopenic events. As patients with cTTP have mild hemolytic anemia, they may remain undetected and undiagnosed. In female patients with cTTP, pregnancy provides a unique opportunity for detecting TTP signs and symptoms [78]. One Japanese male patient was diagnosed with TTP for the first time at the age of 63 [79].

### Diagnosis

cTTP is suspected if the patient presents with thrombocytopenia and ADAMTS13 activity < 10% in the absence of ADAMTS13 inhibitors. However, it is not always technically feasible to determine whether the patient is positive or negative for ADAMTS13 inhibitors. In certain situations, differentiation between congenital and iTTP can only be made after monitoring ADAMTS13 activity and testing the ADAMTS13 activity of the patient’s parents. *ADAMTS13* gene analysis is required for the definitive diagnosis of cTTP. Because asymptomatic parents of a patient with cTTP have heterozygous mutations, their ADAMTS13 activities often range from 30 to 50% [6, 80].

cTTP should be differentiated from ITP that is refractory to common medical management, as well as from pregnancy-induced, complement-mediated TMA and HELLP syndrome. The physician should note that although cTTP always involves severely decreased ADAMTS13 activity, thrombocytopenia and other typical signs and symptoms of TTP may not always be detectable in patients without acute attacks.

### ADAMTS13 gene analysis

cTTP is an autosomal recessive disorder involving homozygous or compound heterozygous *ADAMTS13* gene mutations [5, 81]. Of 65 Japanese patients with genetically confirmed cTTP, 11 and 54 had homozygous and compound heterozygous *ADAMTS13* gene mutations, respectively.

### Treatment

The FFP transfusion regimens that are effective in patients with cTTP vary depending on their conditions; some patients require chronic treatment with periodic FFP transfusions, while others require episodic FFP transfusions only when their condition worsens. Determination of the FFP volume and transfusion duration should take into consideration platelet counts and occult blood scores.

#### FFP transfusion (grade of recommendation: 1B)

An FFP volume of 5–10 mL/kg is empirically transfused once every 2–3 weeks [81]. At the time of onset, the physician should transfuse FFP at a volume of 10 mL/kg and monitor the clinical impact. To minimize the risks of allergy, anaphylaxis, disease transmission, and other adverse reactions to FFP infusion, efforts should be made to collect FFP from the smallest possible number of donors. Antihistamines and corticosteroids may sometimes be used to prevent allergic reactions to FFP, but there is a paucity of scientific evidence to support these treatments. The TTP treatment guidelines of the International Society on Thrombosis and Hemostasis (ISTH) recommend that 10–15 mL/kg of FFP be administered once every 1–3 weeks [82]. However, this recommendation may be suboptimal in Japanese outpatient clinical settings, as the suggested FFP dosage could be excessive for most Japanese patients. Currently, the amount of FFP necessary to prevent organ damage on a long-term basis is unknown [83].

### Classification by severity

Table 5 summarizes the cTTP severity classification scheme used in designated intractable/rare diseases and established by the Japanese MHLW for a medical expense support system. All cases of cTTP are covered by this system, except for mild cases that require no medical treatment.

**Table 5:** cTTP severity classification scheme

1. Severe
Patients undergoing maintenance dialysis and patients with sequelae of stroke or other thrombotic events
2. Moderate
Patients who require fresh frozen plasma transfusion either periodically or episodically
3. Mild
Patients under observation without treatment

### Aggravating factors

Factors that trigger TTP attacks include persistence of the patent ductus arteriosus after birth [77], viral or bacterial infections, pregnancy, and excessive alcohol intake [84]. In particular, pregnancy-induced TTP attacks exert a significant clinical impact not only on mothers but also on their fetuses. A 50% fetal mortality rate was reported for pregnant patients without regular prophylactic FFP infusions [85]. Placental hypoplasia and disturbed fetal blood flow have been suggested as causative factors [86]. Regular prophylactic FFP infusions should be administered in pregnant patients even if they were not needed before pregnancy. Maternal platelet counts and lactate dehydrogenase levels, as well as fetal growth, should be carefully monitored. Regular FFP infusions of ≥ 5 mL/kg once every week are necessary for the management of pregnancy [85].

### Treatment outcome

Appropriate diagnosis and treatment with FFP transfusion will generally achieve excellent results in patients with cTTP. In Japan, 10 deaths were documented among 70 patients diagnosed with cTTP. Five of them died after the start of hemodialysis treatment. These cases suggest that preventing renal deterioration may improve outcomes. It has been suggested that when FFP administration leads to anti-ADAMTS13 alloantibody formation in patients with cTTP, the clinical effects of FFP are compromised. Although no such cases have been reported to date in Japan, this possibility underscores the importance of performing regular ADAMTS13 inhibitor assays.

## Reference materials

### Clinical questions (CQs) related to rituximab

#### Evidence Collection Methods

Primary and Secondary Literature Search on PubMed® (U.S. National Library of Medicine).

As of January 7, 2022, PubMed contained 13,015 and 27,340 articles on TTP and rituximab, respectively. Among these, 335 English-language articles that discussed the effects of rituximab on TTP between 2012 and 2021 were identified. These included 11 clinical guidelines, 43 clinical trial reports, 8 systematic reviews, 151 case reports and 122 others. The guidelines, clinical trial reports, and systematic reviews identified by the primary literature search were screened and reviewed to collect the body of evidence on the effects of rituximab on iTTP.

#### Acute phase

CQ1: Is rituximab recommended for the treatment of acute-phase iTTP?

Answer: Rituximab may be considered for the treatment of acute-phase iTTP (off-label use in Japan, grade of recommendation: 2B)

##### Commentary

Plasma exchange and corticosteroid therapy are the standard of care for initial or recurrent acute events in iTTP. In the 2020 ISTH guidelines for the treatment of TTP, the addition of rituximab to the standard of care is a conditional recommendation for patients with acute TTP events. Multiple single-arm trials reported that concomitant use of rituximab in patients with acute TTP reduced the risk of relapse [49, 87].

In 2009, the prospective, randomized controlled STAR trial was designed to evaluate the efficacy of add-on rituximab to the standard therapy (plasma exchange and corticosteroids) in patients with acute TTP events. This investigator-initiated trial was started after the approval by the institutional review board of a U.S. public research institution. However, this trial was prematurely terminated after three patients were enrolled in 6 months. This study was later redesigned and implemented as the retrospective ReSTAR trial (*n* = 113), which showed that add-on rituximab was associated with a lower relapse rate [88].

In 2011, Scully and a group of U.K. clinicians reported the results of a prospective phase II trial of add-on rituximab in 40 patients with acute iTTP [49]. In this study, patients started rituximab within 3 days of admission at a dosage of 375 mg/m^2^ once weekly for 4 weeks. Rituximab was administered along with plasma exchange and corticosteroid therapy. Compared with historical controls (*n* = 40), the study group had a significantly lower relapse rate (57% vs. 10%), with a median time to relapse of 27 months. Moreover, rituximab shortened the length of hospital stay.

In a study reported in 2017, Chen and other Chinese researchers administered rituximab in addition to plasma exchange and corticosteroids to 14 patients with acute iTTP [87]. Rituximab was given at 375 mg/m^2^ once weekly for 4 weeks. Overall, patients received five plasma exchange sessions (median). They achieved hematological remission at a median of 15 days.

In 2019, Zwicker and American colleagues reported the efficacy results of adjuvant low-dose (100 mg/week) rituximab and plasma exchange in 19 patients with acute iTTP [89]. The rationale behind this study was that the effective dose of rituximab could be lower for immune thrombocytopenia and other autoimmune conditions than for malignant lymphoma. Fixed rituximab doses of 100 mg were administered once weekly for 4 weeks. Compared with the standard dosing regimen, this lower-dose regimen yielded similar outcomes in platelets, B lymphocytes, and ADAMTS13 activity.

In the 2020 ISTH guidelines for the treatment of TTP, the addition of rituximab to plasma exchange and corticosteroid therapy is recommended for iTTP patients experiencing their first acute event [82]. In a French multicenter study published in 2021, a triplet regimen of plasma exchange, immunosuppression with corticosteroids and rituximab, and caplacizumab achieved favorable outcomes in acute-phase patients [90].

In Japan, there is a paucity of data on the efficacy and safety of rituximab in acute-phase patients. In light of available overseas clinical study data and treatment guidelines, add-on rituximab may be considered for the treatment of acute-phase iTTP on a case-by-case basis (grade of recommendation: 2B).

#### Patients with refractory or early-relapse TTP

CQ2: Is rituximab recommended for the treatment of refractory or early-relapse patients with iTTP?

Answer: Add-on rituximab is recommended for the treatment of refractory or early relapse patients with iTTP (grade of recommendation: 1B).

##### Commentary

The addition of rituximab is recommended for the treatment of recurrent or refractory TTP events by the 2020 ISTH guidelines [82] and the consensus report of Australian and New Zealand experts on the management of TMA [91]. iTTP is a rare disease, and no randomized controlled trials of rituximab in relapsed or refractory iTTP have been conducted or published. Well-designed, open-label Phase II studies of add-on rituximab have been conducted, and the results have typically shown lower relapse rates compared with historical controls. The time to platelet count recovery and the length of hospital stay decreased with the addition of rituximab, although its effects varied between studies.

In a 2006 U.K. study by Scully et al., 25 patients with relapsed or refractory disease were treated with rituximab 375 mg/m^2^ once weekly for 4 weeks in addition to plasma exchange and corticosteroids [92]. All 25 patients achieved complete clinical and laboratory remission after a median of 11 days of rituximab therapy, with 23 showing no evidence of ADAMTS13 inhibitors.

In 2012, Froissart and other researchers affiliated with the French Thrombotic Microangiopathies Reference Center published the results of a prospective phase II trial of add-on rituximab in 22 refractory or recurrent cases [50]. Four 375 mg/m^2^ doses of rituximab were administered over 15 days. Compared with historical controls, the study patients had a significantly shorter time to platelet count normalization, and none relapsed within 1 year.

In 2015, Clark and other Canadian clinicians reported the results of a prospective phase II trial of add-on rituximab in 20 refractory and 20 relapsed patients [93]. Rituximab was given at 375 mg/m^2^ once weekly for 4 weeks. At week 8, complete response was documented in 74% (14/19) of refractory patients and in 89% (16/18) of relapsed patients. One-year survival rates were 100% and 85% in relapsed and refractory patients, respectively.

In Japan, Miyakawa and colleagues conducted an investigator-initiated Phase II trial funded by the Health and Labour Sciences Research Program, and the results were published in 2016 [51]. In this study, patients with platelet counts ≤ 50 × 10^9^/L after five plasma exchange sessions or with ADAMTS13 inhibitor titers ≥ 2 BU/mL were defined as refractory. Seven refractory patients were administered rituximab 375 mg/m^2^ once weekly for 4 weeks in addition to plasma exchange and corticosteroids. At 4 weeks of study treatment, platelet counts exceeding 150 × 10^9^/L and 100 × 10^9^/L were documented in 33% (2/6) and 83% (5/6) of patients, respectively.

**Fig. 1:**
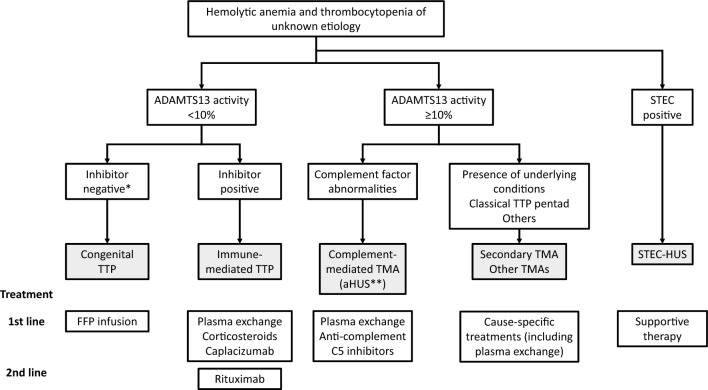
Diagnostic and treatment algorithm for TMA. (Asterisk) The population of patients with iTTP includes a group of patients who are negative for ADAMTS13 inhibitors but positive for ADAMTS13-binding antibodies. (Double asterisks) The term “atypical HUS” (aHUS) is sometimes used conventionally in health insurance and other documentation. TMA thrombotic microangiopathy, TTP thrombotic thrombocytopenic purpura, ADAMTS13 a disintegrin-like and metalloprotease with thrombospondin type 1 motif 13, aHUS atypical hemolytic uremic syndrome, FFP fresh frozen plasma, STEC Shiga toxin-producing *Escherichia coli*

In 2016, Benhamou and other researchers affiliated with the French Thrombotic Microangiopathies Reference Center published the results of a prospective phase II trial of add-on rituximab in 24 refractory patients [94]. Two 375 mg/m^2^ doses of rituximab were infused within 14 days of admission, and an additional dose was administered if peripheral B lymphocytes were detectable on day 15. Two to three doses of rituximab achieved similar results in terms of ADAMTS13 activity, ADAMTS13 inhibitor levels, and 1-year relapse compared with the historical control group that received the standard four-dose regimen Fig. 1.

Rituximab is recommended for the treatment of relapsed or refractory TTP because multiple open-label Phase II trials have demonstrated the efficacy and safety of rituximab for this indication (grade of recommendation: 1B). No randomized controlled studies have been conducted to evaluate rituximab for this indication. This therapy is covered by Japan’s current national health insurance system.

#### Remission period

CQ3: Is rituximab recommended if a patient with iTTP shows a marked reduction in ADAMTS13 activity during remission?

Answer: If ADAMTS13 activity decreases to < 10% during remission in a patient with iTTP, rituximab may be considered for preventing clinical relapse (off-label use in Japan, grade of recommendation: 2B).

##### Commentary

Approximately 30% of patients with iTTP relapse after their platelet counts return to normal levels by plasma exchange. Although patients in remission may not have abnormal platelet counts or other conditions that suggest relapse, their risk of relapse could increase if ADAMTS13 activity falls to < 10%.

In the 2020 ISTH guidelines for the treatment of TTP, the use of rituximab is suggested to be superior to its nonuse for remitted patients who still have low ADAMTS13 activity [82]. This suggestion is based on the findings that patients receiving rituximab have fewer relapses and longer relapse-free survival. According to the ISTH's Good Practice Statements for the Clinical Care of Patients with TTP, patients in remission should typically be assessed every month for the first 3 months, then every 3 months for the first year, then every 6–12 months if stable [95].

In 2012, a group of U.K. researchers led by Westwood published the results of a retrospective study analyzing 15 patients with remitted iTTP [96]. These patients repeatedly had severely reduced ADAMTS13 activity levels, and were given rituximab as prophylaxis to prevent relapse. ADAMTS13 activity normalized within 3 months in 14 patients (93.3%), and follow-up to a median of 23 months detected a single acute TTP relapse that occurred at 70 months (6.7%). However, four patients required further rituximab courses following a drop in their ADAMTS13 activity levels.

In 2014, Hie and colleagues affiliated with the French Thrombotic Microangiopathies Reference Center presented the results of a retrospective study of 385 patients with iTTP, including 48 patients whose ADAMTS13 activity fell to < 10% during remission [97]. Of these, 30 received preemptive rituximab while 18 did not. After a median of 17 months of follow-up, the relapse incidence among patients treated with rituximab decreased from 0.57 to 0 episodes/year. Moreover, rituximab therapy prolonged relapse-free survival. Three months after the first rituximab infusion, median ADAMTS13 activity returned to 46% (interquartile range, 30–68%). Nine patients (30%) required additional courses of rituximab.

In 2018, Jestin and other clinicians associated with the French Thrombotic Microangiopathies Reference Center published the results of a prospective phase II trial of 92 patients with TTP who developed severe ADAMTS13 deficiency during remission [98]. These patients had a severe decrease in ADAMTS13 activity to < 10% during remission, and received one to four 375 mg/m^2^ doses of rituximab at the discretion of their physician. Preemptive rituximab treatment decreased the median cumulative relapse incidence from 0.33 to 0 episodes/year. Recovery of ADAMTS13 activity was sustained in 34 patients (37%) during a median follow-up of 31.5 months. However, four patients required further rituximab courses following a drop in their ADAMTS13 activity levels. Although severe ADAMTS13 deficiency recurred in 45 patients (49%) after initial improvement, ADAMTS13 activity typically improved with additional courses of preemptive rituximab. Preemptive rituximab prevented clinical relapse in 85% of the study patients.

In 2017, Westwood and British colleagues released the results of a retrospective study of rituximab prophylaxis administered in 76 episodes to 45 patients [99]. In these patients, ADAMTS13 activity dropped from the normal range to ≤ 15%. Four once-weekly doses of rituximab were administered at three dosage levels: standard (375 mg/m^2^), reduced (200 mg), and intermediate (500 mg). Rituximab normalized ADAMTS13 activity in 78.9% of patients. Over a median of 15 months of follow-up, relapse was noted in only three patients (3/19, 15.8%) in the reduced dose group. At a median of 17.5 months after initial prophylaxis, 50% of patient episodes required rituximab re-treatment. Re-treatment occurred at a higher rate in the reduced- vs standard-dose group (0.38 vs 0.17 episodes per year, respectively), suggesting the superiority of the standard regimen (four 375 mg/m^2^ doses).

A marked decrease in ADAMTS13 activity to < 10% during remission suggests a high risk of clinical relapsing within months. Patients presenting with recurrent iTTP can be saved by the standard-of-care therapy, although a certain proportion may die despite treatment or suffer from sequelae. In addition, plasma exchange is associated with risks of allergic reactions and catheterization complications. Given these circumstances, rituximab prophylaxis can be a viable option to consider on a case-by-case basis (grade of recommendation: 2B).
